# Development of a Rapid and Efficient RPA-CRISPR/Cas12a Assay for *Mycoplasma pneumoniae* Detection

**DOI:** 10.3389/fmicb.2022.858806

**Published:** 2022-03-15

**Authors:** Feina Li, Jing Xiao, Haiming Yang, Yao Yao, Jieqiong Li, Huiwen Zheng, Qian Guo, Xiaotong Wang, Yuying Chen, Yajie Guo, Yonghong Wang, Chen Shen

**Affiliations:** ^1^Laboratory of Respiratory Diseases, Beijing Key Laboratory of Pediatric Respiratory Infection Diseases, Beijing Pediatric Research Institute, Beijing Children’s Hospital, Capital Medical University, Key Laboratory of Major Diseases in Children, Ministry of Education, National Clinical Research Center for Respiratory Diseases, National Center for Children’s Health, Beijing, China; ^2^Department of Respiratory Diseases II, Beijing Children’s Hospital, National Clinical Research Center for Respiratory Diseases, National Center for Children’s Health, Capital Medical University, Beijing, China; ^3^Department of Respiratory Diseases I, Beijing Children’s Hospital, National Clinical Research Center for Respiratory Diseases, National Center for Children’s Health, Capital Medical University, Beijing, China

**Keywords:** *Mycoplasma pneumoniae*, recombinase polymerase amplification, RPA, CRISPR/Cas12a, visual detection

## Abstract

*Mycoplasma pneumoniae* (MP) is a one of most common pathogen in causing respiratory infection in children and adolescents. Rapid and efficient diagnostic methods are crucial for control and treatment of MP infections. Herein, we present an operationally simple, rapid and efficient molecular method for MP identification, which eliminates expensive instruments and specialized personnel. The method combines recombinase polymerase amplification (RPA) with clustered regularly interspaced short palindromic repeats (CRISPR)/CRISPR associated proteins (Cas) 12a-based detection, with an optimal procedure less than 1 h from sample to result including DNA extraction (25 min), RPA reaction (39°C for 15-20 min), CRISPR/Cas12a detection (37°C for 10 min) and visual detection by naked eyes (2 min). This diagnostic method shows high sensitivity (two copies per reaction) and no cross-reactivity against other common pathogenic bacteria. Preliminary evaluation using 201 clinical samples shows sensitivity of 99.1% (107/108), specificity of 100% (93/93) and consistency of 99.5% (200/201), compared with real-time PCR method. The above data demonstrate that our developed method is reliable for rapid diagnosis of MP. In conclusion, the RPA-CRISPR/Cas12a has a great potential to be as a useful tool for reliable and quick diagnosis of MP infection, especially in primary hospitals with limited conditions.

## Introduction

*Mycoplasma pneumoniae* (MP) is a major causative agent of community-acquired pneumonia in humans, especially children and adolescents, accounting for up to 40% of cases (Leal et al., 2020; Xue et al., 2020; Kakiuchi et al., 2021). MP infections occur throughout the year with epidemic peaks at intervals of 3-7 years (Atkinson et al., 2008; Rivaya et al., 2020; Jiang et al., 2021). The clinical manifestations of MP infection lack specificity, and the course is ordinarily mild and self-limiting, however, up to 25% of patients can develop severe pneumonia or even extra-pulmonary complications (Wang et al., 2019b,c; Chen Y. C. et al., 2020; Meyer Sauteur et al., 2021a).

The diagnostic methods of MP infection include culture-based method, serological test and molecular diagnosis (Waites et al., 2017). Culture-based method offers an irrefutable evidence for MP infection, serological test is most commonly used to detect MP in primary hospitals for its convenience, and molecular approaches, especially real-time PCR, are currently considered as powerful diagnosis tools in clinical laboratories due to its obvious advantages such as high sensitivity and specificity. The high morbidity of MP infections reinforces the need for timely diagnosis. While culturing of MP is time-consuming and relatively insensitive due to its slow growth and fastidious nature (Leal et al., 2020). Reliable results of serological test rely on a single high antibody titer, or ≥ 4-fold increase in antibody titers of paired acute-phase and convalescent-phase serums, nevertheless, the former often exhibits false-negative result in the early stages of MP infection due to multiple factors, and the later requires 2-4 weeks of monitoring, which delays diagnosis and complicates the clinical course (She et al., 2010; Kishaba, 2016). Molecular diagnostic tests such as real-time PCR (Zhao et al., 2012) are generally performed in the central laboratories with specific equipment and skilled operators, which remain difficult to implement in grass-root laboratories and point-of-care detection (Schmitt et al., 2013; Yuan et al., 2018). Due to the unmet clinical needs, it is urgent to develop rapid, reliable, easy-to-use and low-cost diagnostic technologies for MP detection. In recent years, many isothermal amplification techniques are developed for on-site, point-of-care and *in situ* assay applications (Reid et al., 2018), like loop-mediated isothermal amplification (LAMP; Cai et al., 2019), multiple cross displacement amplification (MCDA; Wang et al., 2020), dual-priming isothermal amplification (DAMP; Ding et al., 2019) and recombinase polymerase amplification (RPA; Fan et al., 2020).

RPA technology was developed in 2006 by Piepenburg et al. (2006), and generally performed within less than 30 min at 22-45°C (with optimum temperature 37-42°C; Silva et al., 2015; Clarke et al., 2016; Tu et al., 2017; Lobato and O’Sullivan, 2018). Therefore, RPA eliminates thermal cycles and reduces equipment requirements, which enables detection in low-resource settings. RPA assay has been demonstrated to tolerate certain inhibitors and show high sensitivity and specificity (Lobato and O’Sullivan, 2018). Currently, RPA assay has been widely applied for target amplification in many fields, including pathogen diagnosis, food safety test, synthetic biology and mutagenesis (Li J. et al., 2018).

Recently, clustered regularly interspaced short palindromic repeats (CRISPR)/associated proteins (Cas) systems provide a new and promising approach for nucleic acid diagnostics, as the class II type V and VI Cas proteins, such as Cas12a, Cas12b, Cas13a, and Cas14, are discovered with the collateral cleavage activity (Shmakov et al., 2015; Peng et al., 2020). Cas12a (previously called Cpf1) is a RNA-guided endonuclease with cis- and trans-cleavage DNase activities (Zetsche et al., 2015; Li S. Y. et al., 2018). After recognizing targeted DNA sequences that are complementary to the single CRISPR RNA (crRNA) and juxtaposed with a protospacer adjacent motif (PAM), Cas12a produces cis-cleavage activity and cleaves targeted double-stranded DNA (dsDNA) (Swarts, 2019). Upon releasing the PAM-distal ends of cleaved dsDNA targets and generating sticky ends, the ternary complex of Cas12a/crRNA/targeted dsDNA yields collateral trans-cleavage activity and indiscriminately degrades nearby non-targeted single-stranded DNA (ssDNA) with thousands of turnovers per second (Swarts and Jinek, 2019). Due to PAM-dependent target DNA recognition and effective amplification of signals, CRISPR/Cas12a detection has high specificity and sensitivity. Additionally, cleavage results of CRISPR/Cas12a system can be visualized using lateral flow biosensor (LFB) or fluorescence reader by introducing ssDNA reporter labeled with fluorophore and biotin or alternatively with fluorophore and quencher into an *in vitro* reaction (Xiong et al., 2020; Zhu et al., 2021).

Now, CRISPR/Cas12a detection integrated with RPA has been successfully applied in rapid and accurate detection of various pathogens including viral pathogens (such as SARS-CoV-2, human *metapneumovirus*, human *papillomavirus* and African swine fever; Bai et al., 2019; Yin et al., 2020; Qian et al., 2021; Sun et al., 2021; Talwar et al., 2021), fungal pathogens (for example *Elsinoë fawcettii*; Shin et al., 2021), Bacterial pathogens (such as *Escherichia coli*, *Listeria monocytogenes*, *Staphylococcus aureus*, *Vibrio parahaemolyticus*, *Salmonella* sp., *Xanthomonas arboricola* and *Pseudomonas aeruginosa*; Chen Y. et al., 2020; Cai et al., 2021; Liu et al., 2021; Luo et al., 2021). In this study, RPA coupled with CRISPR/Cas12a (RPA-CRISPR/Cas12a) has been developed for MP detection. This two-step method includes amplification of the *P1* gene using RPA within 15-20 min at 39°C and CRISPR/Cas12a detection within 10 min at 37°C. Detection results can be observed by naked eyes under blue light, and this assay eliminates the requirements of special technical expertise and sophisticated instruments. Except for the analytical sensitivity and specificity, the feasibility of this method has been evaluated using 201 clinical samples. Our developed assay brings a rapid, reliable, sensitive and affordable diagnostic tool for detection of MP, which is suitable for use in primary hospitals.

## Materials and Methods

### Materials

Recombinase polymerase amplification Kits used for isothermal amplification were purchased from Lesunbio Co., Ltd. (Wuxi, China). Primers for RPA reaction, crRNA and double distilled water were ordered from Sangon Biotech Co., Ltd. (Shanghai, China). The ssDNA was synthesized by TianyiHuiyuan Biotechnology Co., Ltd. (Beijing, China). The 100 bp DNA ladder was obtained from TransGene Biotech Co., Ltd. (Beijing, China). EnGen^®^ Lba Cas12a (Cpf1) and NEBuffer™ r2.1 were purchased from New England BioLabs (Beijing, China). QIAamp DNA Mini Kits used for DNA extraction were obtained from Qiagen GmbH (Hilden, Germany).

### Bacterial Strains and Clinical Specimens

The MP reference strain (M129), five clinical isolates of MP and 14 strains of common pathogenic bacteria were obtained from American Type Culture Collection (ATCC) and Beijing Children’s Hospital (BCH; Table 1). After incubation with 20 μL proteinase K and 200 μL buffer AL at 56°C for 10 min, genomic DNA was extracted and purified from those strains using the QIAamp DNA Mini Kits according to the commercial instruction. A total of 201 clinical samples (128 were oropharyngeal swab, 40 were bronchoalveolar lavage fluid and 33 were sputum) were collected from suspected cases of MP infection at BCH. These respiratory samples were tested by real-time PCR with commercially available diagnostic kits (Mole Bio-Science Co., Ltd., Jiangsu, China) on the Agilent Mx3005P platform. Among them, 108 clinical samples were tested positive for MP infection. After clinical and laboratory diagnoses, DNA templates were extracted from respiratory samples using the method described above. The extracted DNA samples were stored at −20°C before use.

**TABLE 1:** Bacterial strains used in this study.

Bacteria	Strain No. (source of strains)^a^	No. of strains	Diagnostic results of RPA-CRISPR/Cas12a assay^b^
*Mycoplasma pneumoniae*	M129 (ATCC)	1	P
	Isolated strains (BCH)	5	P
*Klebsiella pneumoniae*	ATCC 10031	1	N
	ATCC 700603	1	N
	Isolated strain (BCH)	1	N
*Haemophilus influenzae*	Isolated strain (BCH)	1	N
*Acinetobacter baumannii*	ATCC 19606	1	N
	Isolated strain (BCH)	1	N
*Streptococcus pneumoniae*	Isolated strain (BCH)	1	N
*Staphylococcus aureus*	Isolated strain (BCH)	2	N
*Pseudomonas aeruginosa*	Isolated strain (BCH)	1	N
*Group B Streptococcus*	Isolated strain (BCH)	1	N
Carbapenem-resistant *Enterobacter* sp.	Isolated strain (BCH)	1	N
*Escherichia coli*	ATCC 25922	1	N
*Enterococcus faecalis*	ATCC 29212	1	N

*^a^ATCC, American type culture collection; BCH, Beijing children’s hospital. ^b^P, positive; N, negative.*

### Primers and CRISPR RNA Design

The *P1* gene sequences available for MP strains were downloaded from NCBI database^1^ to obtain conserved regions. The primers were designed to amplify a fragment of 100-200 bp within the conserved region according to the principle of RPA primer (Piepenburg et al., 2006; Liu et al., 2016). The primer hairpin and dimer were analyzed based on OligoEvaluator online software^2^. The specificity of the RPA primers was analyzed using the Primer-BLAST of NCBI. The DNA template of M129 was used for primer confirmation.

The PAM recognition and targeted DNA binding were indispensable for Cas12a activation. The crRNA for Cas12a was design to target the region adjacent to the PAM site in amplified fragment following the design principle.

### The Standard Recombinase Polymerase Amplification Reaction

The RPA assay was carried out in 25 μL volumes using a commercial RPA kit at the recommended temperature of 39°C. Briefly, the reaction mixture consisting of 12.5 μL reaction buffer, 1 μL forward primer (10 μM), 1 μL reverse primer (10 μM) and 8 uL double distilled water, was added to a tube containing lyophilized RPA enzyme mix to fully dissolve the contents. Then 1 μL template and 1.5 μL magnesium acetate were added before incubated at 39°C for 20 min. The RPA products were verified by electrophoresis on a 2.5% agarose gel. Images were taken using an Imaging System (Gel Doc XR +, Bio-Rad, America).

### Clustered Regularly Interspaced Short Palindromic Repeats/Cas12a Detection

The CRISPR/Cas12a trans-cleavage system contained 2 μL 10 × NEBuffer r2.1, 0.5 μL Cas12a (10 μM), 1 μL crRNA (10 μM), 0.5 μL ssDNA reporter (5′-FAM-TATTATTATTATTT-BHQ1-3′, 10 μM), 14 μL double distilled water and 2 μL RPA product in a final volume of 20 μL. The reaction was performed at 37°C for 20 min, and then the fluorescent signal was examined by Real-Time PCR System (AriaMx, Agilent, America) or naked eyes under blue light using the Imager System.

### Sensitivity and Specificity of the Recombinase Polymerase Amplification-Clustered Regularly Interspaced Short Palindromic Repeats/Cas12a Assay

To determine the sensitivity of the RPA-CRISPR/Cas12a assay, genomic DNA of M129 was subjected to 10-fold serial dilutions to perform the RPA reaction. The DNA copy number was calculated using the following equation: DNA copy number (copy number/μL) = [6.02 × 10^23^ × genomic DNA concentration (ng/μL) × 10^–9^]/[genomic DNA length (nt) × 660]. The specificity of the assay was evaluated using the DNA templates extracted from 5 clinical isolates of MP and 14 strains of common pathogenic bacteria. Subsequently, the CRISPR/Cas12a detection assay was performed against the above RPA products. Each reaction process was repeated three times.

### Feasibility of Recombinase Polymerase Amplification-Clustered Regularly Interspaced Short Palindromic Repeats/Cas12a Assay Using Clinical Samples

Two hundred and one clinical samples were used to evaluate the performance of RPA-CRISPR/Cas12a assay for MP detection. Five μL DNA templates of each clinical sample were added to RPA reaction system. Double distilled water and DNA template of M129 were used as negative control and positive control, respectively. The performance of RPA-CRISPR/Cas12a assay was compared with that of a commercial real-time PCR assay for MP detection.

### Statistical Analysis

The *kappa* and *P* values between real-time PCR and RPA-CRISPR/Cas12a assay were calculated using the chi-square test. SPSS 16.0 software was utilized for statistical analysis, *kappa* value > 0.75 indicated good consistency, and *P* value < 0.05 was considered to be statistically significant.

## Results

### Confirmation and Detection of *Mycoplasma pneumoniae*-Recombinase Polymerase Amplification Reaction

The sequences of forward primers (F1-F4) and reverse primers (R1-R4) were shown in Table 2, and six sets of primers were used for amplifying a 100-200 bp fragment of *P1* gene (Supplementary Table 1). The primers were screened according to the brightness and sharpness of target band on gel electrophoresis. As shown in Supplementary Figure 1, the second set of primers resulted in a strong target band and showed excellent amplification effect. Thus, F1 and R2 were used for RPA reaction (Supplementary Figure 2A), and the target product was 129 bp in length.

**TABLE 2:** Primers and crRNA designed in this study.

Primer/crRNA	Sequence	Length
F1	CTACTGTAACTGGTTGACCATATGCCTTACT	31 nt
F2	CTTTAACAATAACCGCTGGTTTGAATATGT	30 nt
F3	CCTTTAACAATAACCGCTGGTTTGAATATGT	31 nt
F4	CAACAAACAAACTGACGGGTTAAAGGATCTA	32 nt
R1	CAAACCAGCGGTTATTGTTAAAGGGTAGAT	30 nt
R2	CAGCAACTGCCATCCGTGGTACATATTCAAAC	32 nt
R3	GCGCTACTAAGTTCAGGTTGCTTTCAAGTT	30 nt
R4	TAAGTTCAGGTTGCTTTCAAGTTCATCGTA	30 nt
crRNA	UAAUUUCUACUAAGUGUAGAUACAAUAACCG CUGGUUUGAA	41 nt
		

### Visualization of the Clustered Regularly Interspaced Short Palindromic Repeats/Cas12a Detection Results

The crRNA sequence was shown in Table 2, and its target site in RPA product was shown in Supplementary Figure 3. To visualize the non-specifically cleavage activity of CRISPR/Cas12a detection, the ssDNA reporter tagged with FAM and BHQ1 was added to the reaction system and served as the source of fluorescence. When the Cas12a/crRNA complex recognized the amplified target, the ssDNA reporter was cleaved and then generated fluorescence (Supplementary Figures 2B, 4).

### Optimization Conditions for Recombinase Polymerase Amplification-Clustered Regularly Interspaced Short Palindromic Repeats/Cas12a Assay

**FIGURE 1:**
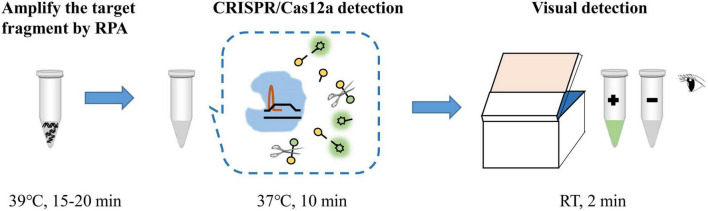
Schematic illustration of RPA-CRISPR/Cas12a assay for detection of MP. RT, room temperature.

**FIGURE 2:**
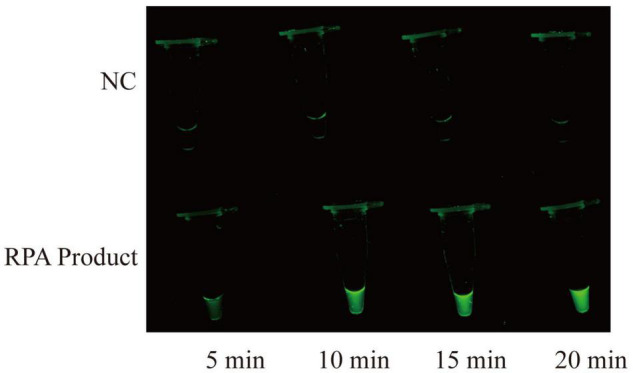
Time optimization for CRISPR/Cas12a assay. CRISPR/Cas12a assay is performed at varying times from 5 to 20 min, and the fluorescence signals are directly observed by naked eyes under blue light. The RPA products are obtained from 2 copies genome. NC, negative control.

The RPA-CRISPR/Cas12a assay was used for detection of MP, and the schematic illustration was shown in Figure 1. To determine the optimal reaction time of CRISPR/Cas12a detection, RPA products amplified from 2 copies genome were performed CRISPR/Cas12a assay with varying incubation time from 5 to 20 min (interval of 5 min). To reduce the use of expensive equipment, fluorescence signals were observed directly by the naked eye under blue light. According to fluorescence intensity at different incubation time, a reaction time of 10 min was recommended for the CRISPR/Cas12a trans-cleavage assay (Figure 2).

### Sensitivity of the Recombinase Polymerase Amplification-Clustered Regularly Interspaced Short Palindromic Repeats/Cas12a Assay

**FIGURE 3:**
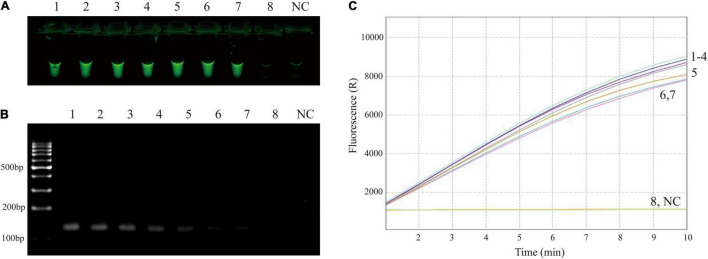
Sensitivity confirmation of RPA-CRISPR/Cas12a assay for MP detection. **(A)** Direct observation by naked eyes under blue light. **(B)** Visualization of RPA products by agarose gel electrophoresis. **(C)** Real-time fluorescence applied for further confirming the results. Tube/signal 1-8 represent template level (genomic DNA of M129) of 2 × 10^6^, 2 × 10^5^, 2 × 10^4^, 2 × 10^3^, 2 × 10^2^, 2 × 10^1^, 2 × 10^0^, 2 × 10^–1^ copies per reaction. NC, negative control.

The sensitivity of RPA-CRISPR/Cas12a assay was determined using M129 genome at various dilutions (ranging from 2 × 10^6^ to 2 × 10^–1^ copies/μL). As shown in Figure 3A, the limit of detection of the RPA-CRISPR/Cas12a assay was 2 copies per reaction. Compared with agarose gel electrophoresis (Figure 3B), CRISPR/Cas12a detection was more sensitive and more convenient to interpret results.

### Specificity of the Recombinase Polymerase Amplification-Clustered Regularly Interspaced Short Palindromic Repeats/Cas12a Assay

**FIGURE 4:**
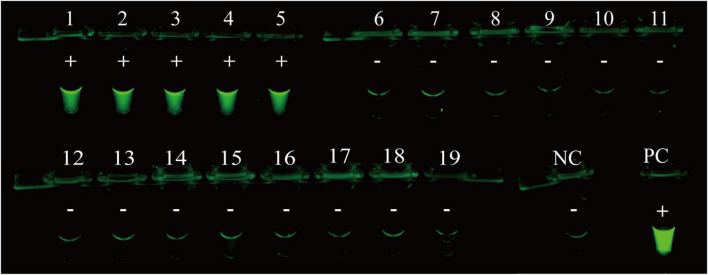
Analytical specificity of RPA-CRISPR/Cas12a assay for MP detection. The RPA-CRISPR/Cas12a assay is conducted using genomic DNA extracted from 19 pathogens. Tube 1-5, clinical isolates of MP; Tube 6-19, *Klebsiella pneumonia* ATCC 10031, *Klebsiella pneumonia* ATCC 700603, *Klebsiella pneumonia* isolated strain, *Haemophilus influenzae* isolated strain, *Acinetobacter baumannii* ATCC 19606, *Acinetobacter baumannii* isolated strain, *Streptococcus pneumoniae* isolated strain, *Staphylococcus aureus* isolated strain, *Staphylococcus aureus* isolated strain, *Pseudomonas aeruginosa* isolated strain, *Group B Streptococcus* isolated strain, isolated strain of carbapenem-resistant *Enterobacter* sp., *Escherichia coli* ATCC 25922, *Enterococcus faecalis* ATCC 29212; PC, positive control; NC, negative control.

The specificity of the RPA-CRISPR/Cas12a assay was evaluated by using templates extracted from 5 MP isolates and 14 non-MP strains. The results of visual detection indicated that 5 MP isolates produced fluorescence signals, whereas negative results were obtained from 14 non-MP strains (Figure 4). Therefore, RPA-CRISPR/Cas12a assay showed no cross-reactions against other common pathogenic bacteria of respiratory infection, suggesting its high specificity (100%).

### Validation of the Recombinase Polymerase Amplification-Clustered Regularly Interspaced Short Palindromic Repeats/Cas12a Assay for *Mycoplasma pneumoniae* Detection Using Clinical Samples

The feasibility of RPA-CRISPR/Cas12a assay for MP detection was assessed using the templates extracted from 201 respiratory samples, and the obtained results were compared with those of real-time PCR as the reference method. Of 108 samples detected positive by real-time PCR, 107 samples were detected positive by RPA-CRISPR/Cas12a assay. Meanwhile, 93 samples detected negative by real-time PCR, were also diagnosed negative by RPA-CRISPR/Cas12a assay. Compared with real-time PCR, the analytical sensitivity and specificity of RPA-CRISPR/Cas12a assay for MP detection were 99.1 and 100%, respectively, and the agreement between these two methods was 99.5%. The *kappa* value was more than 0.75 and *P* value was more than 0.05 (Table 3). Therefore, there was no significant difference between the detection results of RPA-CRISPR/Cas12a and real-time PCR.

**TABLE 3:** The clinical performance of RPA-CRISPR/Cas12a assay for MP detection compared with real-time PCR as the reference method.

Real-time PCR	RPA-CRISPR/Cas12a		Total	Performance of RPA-CRISPR/Cas12a compared with real-time PCR				
			
	Positive	Negative		Sensitivity (%)	Specificity (%)	Accordance rate (%)	*Kappa* value	*P* value
Positive	107	1	108	99.1	100	99.5	0.99	1.00
Negative	0	93	93	–	–	–	–	–
Total	107	94	201	–	–	–	–	–

## Discussion

*Mycoplasma pneumoniae* is an important etiologic agent causing respiratory infections in children and adolescents (Wang et al., 2021). The traditional diagnostic methods for MP detection are underused due to their cost, insensitivity, time-intensive nature or requirements for specialized instruments and significant expertise (Petrone et al., 2015). There is an increasing demand for efficient, portable and affordable diagnostic methods.

In this study, a robust and rapid MP detection method combining CRISPR/Cas12a based detection and the RPA reaction has been established as follow: first, the target sequence is enriched by RPA reaction at one temperature; second, trans-cleavage activity of CRISPR/Cas12a is activated in presence of the target RPA product; Finally, the diagnostic result is judged on the basis of its fluorescence intensity observed by naked eyes under blue light. With the above processes, rapid detection of MP can be effectively achieved.

MP strains harbor multiple genotypes (Xiao et al., 2015; Meyer Sauteur et al., 2021b), and genetic variants within the target region may lead to false negative results for molecular diagnosis (Unckless et al., 2017; Merida-Vieyra et al., 2019; Xue et al., 2020). In previous studies, *P1*, *CARDS toxin*, *ATPase*, *16S rRNA*, *23S rRNA*, *RepMp1* and *RepMP4* have been reported as target sequence (Dumke et al., 2007; Winchell et al., 2008; Zhou et al., 2015; Leal et al., 2020). Among them, *P1* gene has been demonstrated to be high selective and efficient for sequence detection (Merida-Vieyra et al., 2019; Xue et al., 2020). *P1* gene encodes an important 170-kDa protein as the major adhesion protein, which is essential for MP infection and successful colonization. The P1 adhesion protein is a major immunogen of MP, which could be used as a target for development of clinical diagnostic reagents and as a vaccine candidate (Inamine et al., 1988; Kenri et al., 1999). Although similar genes are found in other *Mycoplasma* species, highly conserved regions of this gene are unique to MP (Su et al., 1987; Pitcher et al., 2006). Notably, two-thirds of the *P1* gene sequence is present in multiple copies (Su et al., 1988), thus, *P1* gene is an attractive molecular target. Therefore, the RPA primers are designed for the conserved region of *P1* gene in the present study, and the assay shows no cross-reactivity with common pathogenic bacteria (Figure 4).

Currently, most commercial molecularly based tests generally require more than 60 min to detect MP (Leal et al., 2020). In the present report, RPA reaction could be completed within 15-20 min at 39°C. What’s more, the RPA reaction is performed with commercial kits, and the RPA enzymes are mixed in lyophilized form and stored in reaction tubes, which is convenient to storage and prepare reaction system. Also, previous studies have reported that the RPA can amplify target nucleic acids in the presence of PCR inhibitors (Kersting et al., 2014; Li J. et al., 2018; Lobato and O’Sullivan, 2018). Therefore, the robust RPA assay can be performed efficiently in relatively short time and resource-limited settings.

Clustered Regularly Interspaced Short Palindromic Repeats/Cas is an emerging technology for biological analysis and molecular diagnostics (Hendriks et al., 2020; Xiang et al., 2020). The trans cleavage activity of CRISPR/Cas (such as Cas12a) can be activated specifically by target sequences, which can efficiently cleave ssDNA reporters (thousands of turnovers per second) for further signal amplification (Wang et al., 2019a; Peng et al., 2020). It has been demonstrated that CRISPR/Cas12a-based assay shows a superior sensitivity than the RPA-only detection in general (Xiong et al., 2020). Thus, CRISPR/Cas12a can not only compensate for the non-specific amplification of RPA reaction, but can also improve the sensitivity and efficiency of molecular diagnosis. More notably, the CRISPR/Cas12a assay does not require cumbersome primer design or excessively rigorous experimental operations (Gong et al., 2021), and has emerged as an ideal molecular diagnostic tool. In this study, CRISPR/Cas12a combined with RPA reaction has been successfully established to rapidly detect nucleic acids of MP strains, and this diagnostic approach has single-copy sensitivity (Figure 3) and high specificity (Table 1 and Figure 4).

Visual detection is crucial for molecular diagnostics, especially in the absence of instruments (Xiong et al., 2020). In this report, a fluorescence reporter molecule labeled with a fluorescent dye (FAM) on the 5′ end and a fluorescent quencher (BHQ1) on the 3′ end to visualize the CRISPR/Cas12a detection results. When optimizing the detection conditions, we observe the fluorescence intensity of CRISPR/Cas12a system at different time under blue light, after adding the target products amplified from 2 copies template. When incubation time exceeds 10 min, detection systems produce significant fluorescence (Figure 2). The direct observation enables simple and rapid interpretation of CRISPR/Cas12a detection results with an accuracy comparable to the instrument detection (Figure 3C).

Aside from the high sensitivity and specificity, RPA-CRISPR/Cas12a assay also has good efficiency. The whole process of RPA-CRISPR/Cas12a assay merely requires less than 1 h, including 25 min for rapid template preparation, 15-20 min for RPA reaction, 10 min for CRISPR/Cas12a assay and 2 min for visual detection by naked eyes (Figure 1). Of note, the detection is performed at relatively low constant temperature and does not need sophisticated instruments or skilled personnel, which could significantly improve the detection efficiency and reduce the diagnostic costs. Hence, the RPA-CRISPR/Cas12a assay developed here has great potential for application.

Finally, templates extracted from 201 respiratory samples are used to validate the clinical application of the RPA-CRISPR/Cas12a assay. Consistency analysis (consistency of 99.5%, *kappa* = 0.99) and difference analysis (*P* = 1.00) demonstrate RPA-CRISPR/Cas12a assay has equal ability with real-time PCR in MP detection (Table 3), which indicates that the RPA-CRISPR/Cas12a assay developed in this study is reliable for detection of MP infections in children.

Until now, many new isothermal amplification methods combined with various visualization tools, such as LAMP-LFB (Wang et al., 2019b), MCDA-LFB (Wang et al., 2019c) and real-time RPA assay (Xue et al., 2020), have been applied to detect MP. As summarized in Table 4, RPA just demands a single pair of primers, whereas LAMP and MCDA require 6 and 10 primers, respectively, which illustrates that RPA is more convenient and economical than LAMP and MCDA. Notably, RPA-CRISPR/Cas12a assay can be performed at relatively low temperature even in closed hands or with a hand warmer (Moreno-Mateos et al., 2017; Lobato and O’Sullivan, 2018). Another advantage of RPA-CRISPR/Cas12a assay over MCDA-LFB and LAMP-LFB is its higher sensitivity. In this study, the analytical sensitivity of our established assay is two copies per reaction, while the sensitivity of MCDA-LFB and LAMP-LFB are about 50 and 600 copies per reaction, respectively. Although recombinase-aided amplification (RAA) assay is comparable to RPA-CRISPR/Cas12a assay at the level of sensitivity and efficiency, RPA-CRISPR/Cas12a assay has incomparable advantages over RAA assay in application and promotion, as it eliminates the requirement of special instruments in comparison to fluorescence ration PCR instrument needed for RAA assay. It is noteworthy that RPA-CRISPR/Cas12a assay is available to detect variety of respiratory samples (Table 4). As an assay, our developed approach shows advances in simplicity, sensitivity, efficiency and low-cost.

**TABLE 4:** Comparison of the current approach with other isothermal amplification methods for MP detection.

Approach	No. of primers	Temperature (°C)	Reaction time (min)	Sensitivity (copies per reaction)	Clinical sample^a^	References
RPA-CRISPR/Cas12a assay	2	37/39	27-32	2	201 respiratory specimens (128 swab samples, 40 BALF samples and 33 sputum samples)	This study
RAA assay (fluorescent probe-based method)	2	39	15-30	2.23	311 respiratory specimens (213 BALF samples, 90 sputum samples and 8 swab samples)	Xue et al., 2020
MCDA-LFB assay	10	65	32	∼50 (50 fg)	197 swab specimens	Wang et al., 2019c
LAMP-LFB assay	6	65	62	∼600 (600 fg)	209 swab specimens	Wang et al., 2019b

*^a^BALF, bronchoalveolar lavage fluid.*

We should note that RPA-CRISPR/Cas12a assay has some limitations. Using CRISPR/Cas12a detection increases the risk of cross-contamination due to the necessity to open reaction tubes after the RPA reaction. To avoid false-positive results, CRISPR/Cas12a detection system should be prepared in a separate clean area, and the tube lid should be closed immediately after adding the RPA products. Another smart strategy is developing the two-step method in a single-pot reaction.

## Conclusion

We have selected a suitable target, designed RPA primers and crRNA to successfully establish a reliable RPA-CRISPR/Cas12a assay for detection of MP, and then preliminary validated the feasibility of the proposed method in this study. The obtained data indicate that the developed assay exhibits high sensitivity, specificity and efficiency, and provides a rapid, facile, accurate and affordable method for MP detection. This method is suitable for detection of MP in various respiratory specimens, especially for point-of-care detection in resource-limited areas, which may contribute, to some extent, to control of MP infection.

## Data Availability Statement

The original contributions presented in the study are included in the article/Supplementary Material, further inquiries can be directed to the corresponding author/s.

## Ethics Statement

The studies involving human participants were reviewed and approved by Beijing Children’s Hospital, Capital Medical University. Written informed consent from the participants’ legal guardian/next of kin was not required to participate in this study in accordance with the national legislation and the institutional requirements.

## Author Contributions

FL, JX, and CS conceived and designed the experiments. FL, HZ, QG, XW, YC, and CS performed the experiments. HY and YY contributed the clinical samples. JX, JL, YG, YW, and CS contributed the reagents and materials. FL and CS analyzed the data and wrote the manuscript. All authors have contributed and approved the final version of the manuscript.

## Conflict of Interest

The authors declare that the research was conducted in the absence of any commercial or financial relationships that could be construed as a potential conflict of interest.

## Publisher’s Note

All claims expressed in this article are solely those of the authors and do not necessarily represent those of their affiliated organizations, or those of the publisher, the editors and the reviewers. Any product that may be evaluated in this article, or claim that may be made by its manufacturer, is not guaranteed or endorsed by the publisher.

## Footnotes

1
https://www.ncbi.nlm.nih.gov/nuccore

2
http://www.oligoevaluator.com
