# Supplementary material for: Development of a Rapid and Efficient RPA-CRISPR/Cas12a Assay for Mycoplasma pneumoniae Detection
Source: Front Microbiol. 2022 Mar 15;13:858806. doi: 10.3389/fmicb.2022.858806 (PMC8965353; doi:10.3389/fmicb.2022.858806)
Supplement: Supplementary file 1 [file Data_Sheet_1.docx]

***Supplementary Material***


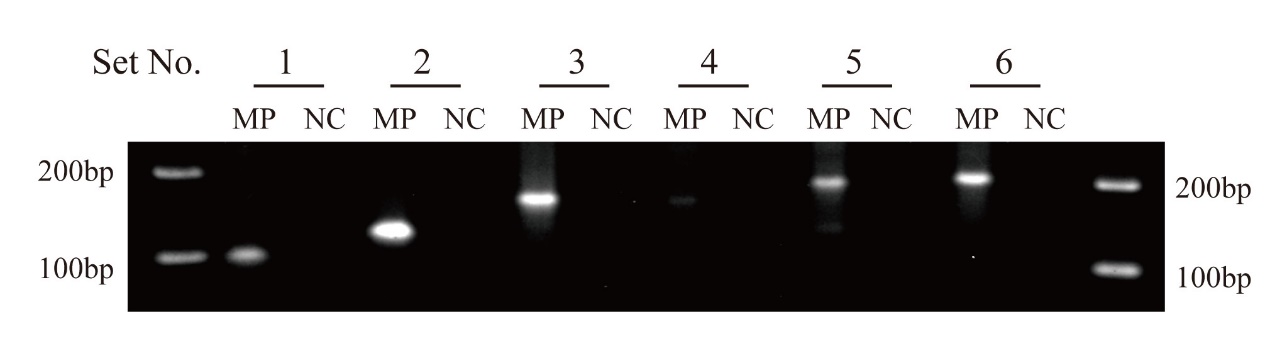


**Supplementary Figure 1** Evaluation of RPA primers by agarose gel electrophoresis. RPA reaction is performed with 6 sets of primers. Set of primers: 1, F1 and R1; 2, F1 and R2; 3, F2 and R3; 4, F3 and R3; 5, F4 and R4; 6, F4 and R3. MP, MP reference strain (M129); NC, negative control.


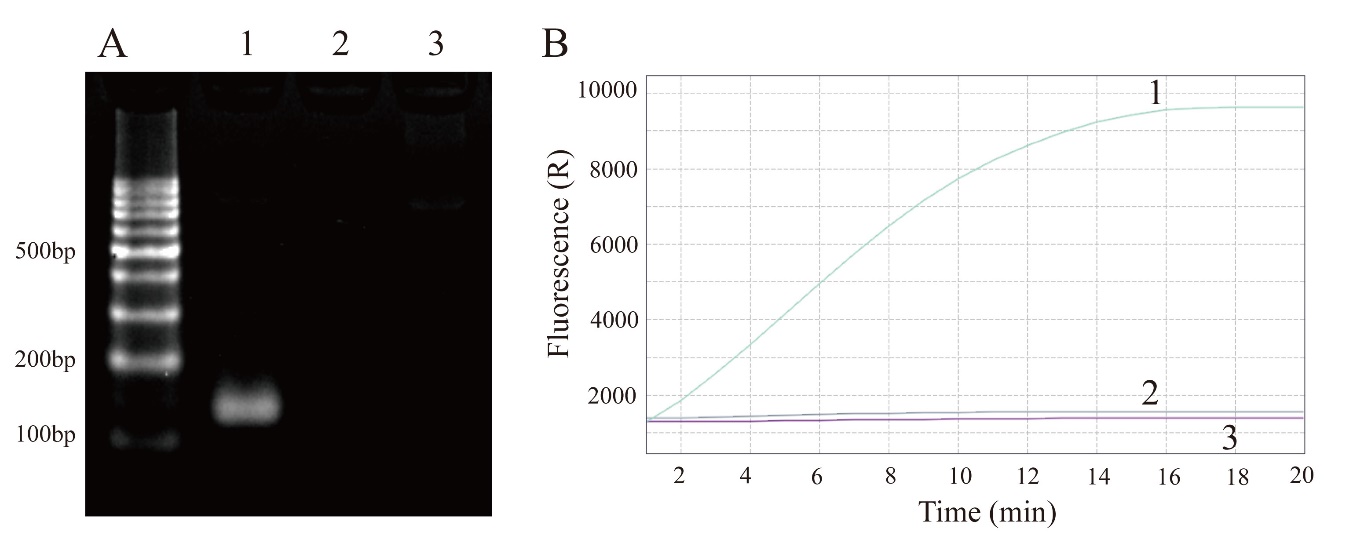


**Supplementary Figure 2** Establishment and confirmation of RPA-CRISPR/Cas12a assay for MP detection. **(A)** RPA products amplified with primers F1 and R2 are detected by agarose gel electrophoresis. **(B)** CRISPR/Cas12a assay is used for detection of the target product. Tube/signal: (1) positive control of MP reference strain (M129); (2) negative control of *Klebsiella pneumonia* ATCC 700603; (3) negative control of double distilled water.


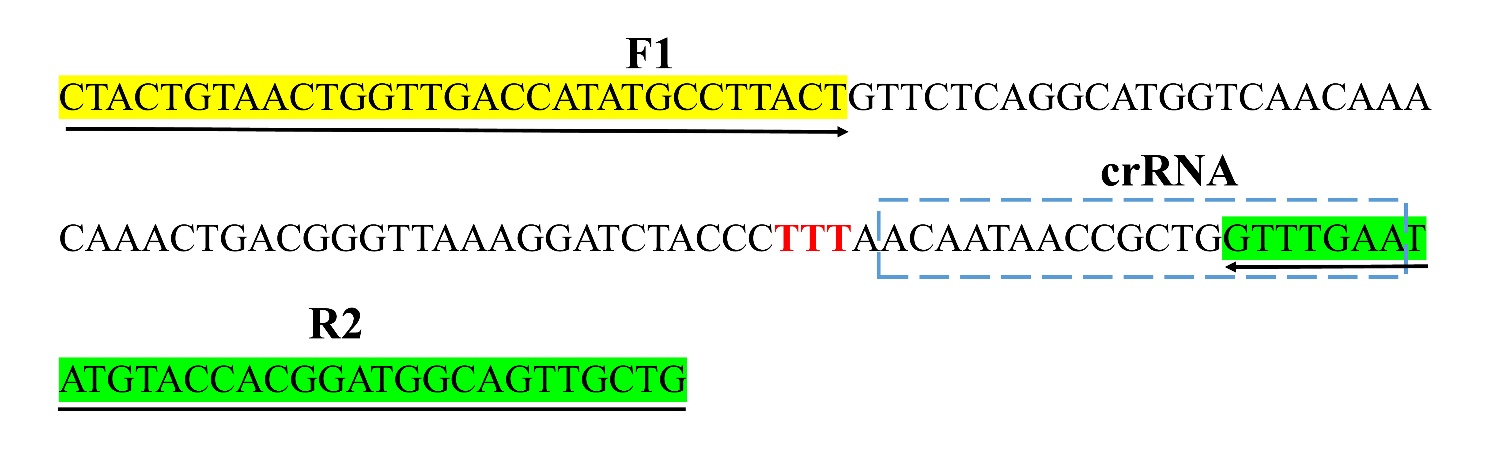


**Supplementary Figure 3** Location of crRNA in the target product. The target site of crRNA is indicated by a blue dashed box, and the PAM sequence is marked in red. The forward primer (F1) and the reverse primer (R2) are highlighted in yellow and green, respectively. The arrows indicate the direction of primers form 5' to 3'.


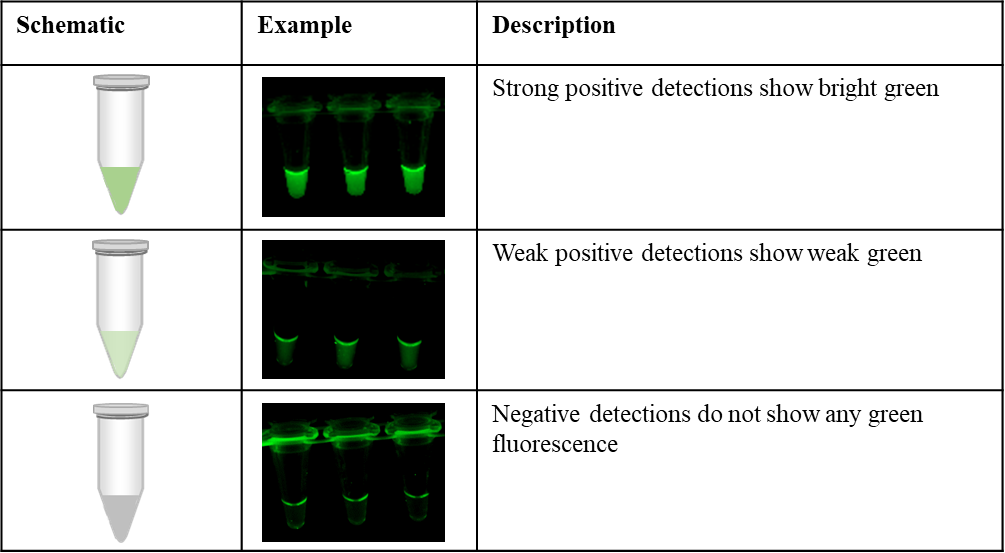


**Supplementary Figure 4** Guidance for interpretation of the visual detection results.

**Supplementary Table 1** Six sets of primers used in this study.

| **Assay** | **Set No.** | **Primers** | **Product size** | **Gene** |
| --- | --- | --- | --- | --- |
| RPA | 1 | F1, R1 | 102 bp | *P1* |
|  | 2 | F1, R2 | 129 bp |  |
|  | 3 | F2, R3 | 167 bp |  |
|  | 4 | F3, R3 | 168 bp |  |
|  | 5 | F4, R4 | 193 bp |  |
|  | 6 | F4, R3 | 200 bp |  |
